# Effect of Dietary Crude Protein Level and Supplemental Herbal Extract Blend on Selected Blood Variables in Broiler Chickens Vaccinated against Coccidiosis

**DOI:** 10.3390/ani8110208

**Published:** 2018-11-15

**Authors:** Anna Arczewska-Włosek, Sylwester Świątkiewicz, Katarzyna Ognik, Damian Józefiak

**Affiliations:** 1 Department of Nutrition Physiology, National Research Institute of Animal Production, 32-083 Balice, Poland; s.swiatkiewicz@izoo.krakow.pl; 2 Department of Biochemistry and Toxicology, Faculty of Biology, Animal Sciences and Bioeconomy, University of Life Sciences in Lublin, 20-950 Lublin, Poland; katarzyna.ognik@up.lublin.pl; 3 Department of Animal Nutrition, Faculty of Veterinary Medicine and Animal Science, Poznan University of Life Sciences, 60-637 Poznan, Poland; damjo@up.poznan.pl

**Keywords:** anticoccidial vaccine, herbal extracts, dietary protein level, blood variables

## Abstract

**Simple Summary:**

Today the poultry industry is facing mounting pressure to phase out chemoprophylaxis. One of the major threats to the poultry production is coccidiosis, a widespread parasitic disease, commonly controlled by in-feed coccidiostats. The use of immunoprophylaxis with the live anticoccidial vaccines instead of coccidiostats in broiler chickens is a promising approach; however, there is a broad reluctance to use them in intensive production due to a possible transient performance deterioration. Thus, nutritional methods such as dietary modification or herbal extracts used as complements to anticoccidial vaccination may support gaining a satisfactory performance, as well as an improved health status in broilers.

**Abstract:**

Immunoprophylaxis with a live anticoccidial vaccine is regarded as the most promising alternative in place of in-feed coccidiostats in the poultry industry. An experiment, designed as a 2 × 2 × 2 factorial arrangement with 6 replicate pens per treatment and 8 male Ross 308 chicks per pen, was conducted to evaluate the effect of dietary crude protein (CP) levels (21.6% or 23.6%, during the starter phase) and a herbal extract (HE) blend dietary supplementation (*Echinacea purpurea*, *Salvia officinalis*, *Thymus vulgaris*, *Rosmarinus officinalis*, *Allium sativum*, *Origanum vulgare*; 0 or 2 g/kg of feed) on selected hematological, biochemical, redox, and immunological parameters in broilers vaccinated against coccidiosis (anticoccidial vaccine (ACV); none or 1× dose, administered at 1 d of age). The blood samples were collected at 14 d of age. Anticoccidial vaccination (*p* < 0.05) had a negative effect on immune responses, as shown by a reduced total white blood cells (WBC) count, a reduced lymphocytes count (L), a higher proportion of heterophils (H) in leukogram assessments, as well as H/L-ratio increase. ACV resulted in a decrease in phagocytic activity assessed as decreased percentage of phagocytic cells, phagocytic index and NBT test, as well as in reductions in plasma glucose and LDL-cholesterol concentrations, and increases in HDL-cholesterol and aspartate aminotransferase (AST) activity. In terms of redox status, ACV significantly increased the catalase (CAT) activity and ferric reducing ability of plasma (FRAP), and decreased malondialdehyde concentrations. An increase in dietary CP in vaccinated chickens resulted in higher relative L and lower relative H counts, a lower H/L ratio, a decrease in AST and an increase in CAT activities, but also a decrease in FRAP and concentrations of lipid peroxides. Vaccinated chickens fed a diet supplemented with HE were characterized by higher relative L and lower relative H counts, a lower H/L ratio, and a decrease in AST activity. A change of diet composition, such as an increase in CP content and dietary HE supplementation, can be recommended in broilers vaccinated against coccidiosis, mainly due to its positive effect in alleviating stress levels. However, the simultaneous increase of CP and HE dietary supplementation offered no additional relevant benefits in most of the blood indices of vaccinated chickens.

## 1. Introduction

Coccidiosis, a parasitic disease caused by the protozoan coccidia of the genus *Eimeria*, is one of the most common intestinal threats to poultry production, causing annual financial losses of over $3 billion globally [1] and €51 million in Poland [2]. The parasites multiply in the intestinal epithelium, leading to diverse effects, from reduced performance and symptoms of a subclinical form of infection such as tissue damage, digestive disorders, malabsorption, and, in cases of severe coccidiosis, death, frequently accompanied by visible clinical signs such as diarrhea and hemorrhage [3,4].

Immunoprophylaxis with a live anticoccidial vaccine (ACV) is regarded as the most promising alternative to in-feed coccidiostats [5]. The vaccine delivers a small number of live virulent or attenuated oocysts which undergo a complete parasitical life cycle in the epithelium. Two to three consecutive infections with circulating oocysts stimulate development of solid immunity [6]. This process sometimes leads to transient performance deterioration by inducing a state of mild subclinical coccidiosis associated with a reduced absorptive intestinal area, malabsorption, and inflammation, as well as secondary enteritis as a result of impaired gut integrity following replication of the vaccine’s oocysts in the intestinal epithelium [7]. Thus, the use of live anticoccidial vaccines in broiler chickens is not widespread, due to the risk of insufficient compensation for reduced body weight gain during the relatively short lifespan of chickens [7].

Numerous recent studies have focused on nutritional methods as complements to immunoprophylaxis, as stated in the review by Arczewska-Włosek and Świątkiewicz [8] on nutrition as a modulatory factor of the efficacy of live ACV in broiler chickens. Moreover, according to Lee et al. [9], a proper protein level in starter diet is crucial to avoid performance deterioration due to vaccination. The authors conducted a series of experiments to examine the effect of various protein levels in starter diets (from 20 to 24%) on the performance of broilers vaccinated with ACV, and the level of 23% or 24% crude protein (CP) yielded a higher body weight gain (BWG) in comparison to 21% and 22% CP. In another study, ACV affected feed intake and caused a reduction in BWG and feed conversion ratio (FCR), independent of dietary CP level; however, the growth rates of chickens fed 21% and 23% CP diets were significantly higher than those fed 19% CP diets, and FCR improved as the CP level increased [10]. In the study by Arczewska-Włosek et al., [11], a higher than normative dietary crude protein content (23.6%) and supplementation with herbal extracts were found to be supportive factors partially limiting the negative influence of live ACV on the performance of broiler chickens, without exerting coccidiostatic properties which could interfere with the circulation of the vaccine strain and thus the acquisition of immunity. Despite numerous reports on the performance results of different nutritional strategies in chickens vaccinated against coccidiosis [7,9,10,11], data concerning their effect on blood variables are limited. The main goal of this study was therefore to evaluate the effects of single and combined nutritional methods, such as increasing dietary CP or/and supplementation with herbal extracts (HE), on selected hematological, biochemical, redox, and immunological blood variables in chickens vaccinated against coccidiosis.

## 2. Materials and Methods

### 2.1. Birds and Experimental Design

The protocols for this study were approved by the 2nd Local Ethical Committee on Animal Testing, Cracow, Poland (Authorization No. 1065/2013). This study was carried out in compliance with Directive 2010/63/EU and in accordance with the Act on Experiments on Animals of 21 January 2005 and the Regulation of the Minister of Science and Information Technology of 29 July 2005 on the National Committee for Animal Experimentation.

A total of 384 one-day-old male Ross 308 broiler chickens, obtained from a commercial hatchery (Daniela Kożuch Poultry Hatchery, Łężkowice, Poland) were randomly assigned to a 2 × 2 × 2 factorial arrangement of treatments to examine the effects of the following factors: ACV (none or 1× dose), dietary CP level (21.6% or 23.6%) and dietary supplementation with a blend of herbal extracts (0 or 2 g/kg feed). Each of the 8 experimental groups comprised 6 replicates (pens) of 8 birds. The birds were kept in standard environmental rearing conditions and in floor pens with wood shavings as a bedding material to ensure the circulation of the vaccine’s oocysts. To reduce the risk of transmission of the vaccine oocysts, the pens were separated by polyvinyl chloride sheets as barriers.

All birds were fed *ad libitum* with maize-soybean meal basal diets free of antibiotic growth promoters and coccidiostats and formulated to meet or exceed the nutrient requirements of broilers for the starter phase [12]. The nutrient content of the diets was calculated according to the chemical composition of raw feedstuffs, and metabolizable energy value according to equations from European Tables [13]. Samples of feed mixtures were analyzed, using standard AOAC methods [14], for moisture (method 930.15), crude protein (method 984.13), crude fat (method 920.39) and ash (method 942.05). Amino acids were analyzed in acid hydrolysates, after initial performic acid oxidation of sulfur amino acids and after alkaline hydrolysis of tryptophan (method 982.30). Calcium content was analyzed by flame atomic absorption spectrophotometry (method 968.08) and total phosphorus content colorimetrically by the molybdovanadate method (method 965.17).

Data relating to performance indices and a profile of oocyst output were previously published and therefore are not presented in this article [11].

### 2.2. Experimental Factors

At 1 d of age, half the chicks were administered *per os* a single dose of commercial live attenuated trivalent anticoccidial vaccine (Livacox^®^ T; BIOPHARM Research Institute of Biopharmacy and Veterinary Drugs, Jílové u Prahy, Czech Republic). The vaccine dose (0.01 mL) contained 300–500 sporulated oocysts of *Eimeria acervulina, Eimeria maxima*, and *Eimeria tenella* suspended in 0.25 mL distilled water. Birds from non-vaccinated groups were administered the same volume of distilled water.

The calculated CP levels in the experimental starter diets were 21.6% and 23.6% in the groups receiving normative or increased CP, respectively, while the respective analyzed values were 21.3% and 23.24% (Table 1).

The HE blend was administered at a dose of 2 g/kg feed (Intermag Ltd., Olkusz, Poland). One kilogram of blend contained: dry extract from *Echinacea purpurea*, 400 mg; oleoresin *Salvia officinalis*, 27,800 mg; oleoresin *Thymus vulgaris*, 5000 mg; oil extract from *Rosmarinus officinalis* 2500 mg; oil from *Allium sativum*, 1670 mg; and oil from *Origanum vulgare*, 1000 mg.

### 2.3. Sample Collection

To evaluate the health status, blood samples were collected at 14 d of age from the wing vein of 6 chickens per experimental group (1 chicken/1 replicate; *n* = 6). After blood collection the chickens were immediately sacrificed by decapitation after electrical stunning.

Hematocrit values (Ht), hemoglobin levels (Hb), and total white blood cells (WBC) with leukograms and red blood cells (RBC) counts were determined using standard methods according to Feldman et al. [15]. The heterophil to lymphocyte ratio (H/L) was also estimated as an indicator of stress condition [16,17].

The immunological analyses involved the identification of the phagocytic activity of leukocytes against the *Staphylococcus aureus* 209P strain, expressed as the proportion of phagocytic cells (%PC) and as the phagocytic index (PI) [18]. The respiratory burst activity of the heterophils was quantified by reduction of nitro blue tetrazolium (NBT) to formazan as a measurement of the production of oxygen radicals [19]. Serum lysozyme concentration was determined by the turbidimetric method [18]. Kits developed by Cormay Co. (PZ Cormay Inc., Lomianki, Poland) were used to determine the following biochemical indices in the blood plasma samples: total protein (TP), uric acid (UA), bilirubin (BIL), creatinine (CREAT), and total cholesterol (TC) and its fractions, i.e., high-density (HDL-C) and low-density (LDL-C) lipoproteins, triacylglycerol (TG), as well as activity of the enzymes alanine aminotransferase (ALT), aspartate aminotransferase (AST), and lactate dehydrogenase (LDH).

As previously described [20], the following indicators of redox status were determined in chicken blood plasma: concentrations of lipid peroxides (LOOH); malondialdehyde (MDA); a ferric reducing ability of plasma (FRAP), as a parameter of antioxidant potential; and activities of superoxide dismutase (SOD) and catalase (CAT).

### 2.4. Statistical Analysis

The data were analyzed using STATISTICA 12 software (Statsoft, Tulsa, OK, United States) software for a three-way ANOVA with ACV, dietary CP level, and HE blend supplementation as the main factors. Duncan’s multiple range test [21] was used to determine the interactions and differences between the treatment’s main effects, considering the probability level of *p* ≤ 0.05 as significant.

## 3. Results

Table 2 and Table S1 in Supplementary Materials present the hematological indices analyzed in blood collected at 14 d of age. A significant increasing effect of HE on RBC count was recorded. The Ht and Hb values were not statistically affected by the experimental factors. The effect of ACV for WBC was statistically significant (*p* ≤ 0.05), as an administration of ACV resulted in a significant decrease in WBC count. Moreover, ACV × CP, CP × HE or ACV × CP × HE interactions were statistically significant (*p* ≤ 0.05) for WBC. In unvaccinated birds, increasing dietary CP levels resulted in increased WBC; however, the combined use of increased CP and HE resulted in WBC levels similar to that obtained in birds receiving diets with normative CP. In vaccinated birds, neither an increase in dietary CP level nor an increase in supplemental HE had an effect on WBC.

When we consider the independent effects, the administration of ACV increased the proportion of H and reduced the proportion of L in leukograms, while the opposite effect was observed by increasing dietary CP. In addition, HE had no effect on these variables (Table 2). ACV × CP, ACV × HE or CP × HE interactions were also found to be significant for proportions (%) of H and L. In vaccinated birds, increased CP or HE supplementation resulted in a reduction of H and in an increase in L proportions in leukograms compared to vaccinated birds with normal CP levels or without HE supplementation, respectively (Table S1 in Supplementary Materials). As a result, a decrease in the heterophils to lymphocytes (H/L) ratio was observed. Moreover, birds receiving a diet with increased CP showed a lower H/L ratio compared to the CPN group. The proportions of monocytes, basophils and eosinophils in leukograms were not significantly affected by the examined variables.

Significant effects of ACV were observed on lysozyme concentration, % PC, PI and NBT; the levels of all these variables were depressed in vaccinated birds. Similarly, HE supplementation resulted in a decrease in lysozyme concentration, % PC and NBT (Table 3 and Table S2 in Supplementary Materials).

In unvaccinated birds, increased CP or HE supplementation resulted in increased AST activities (Table 4, Table S3 in Supplementary Materials). A significant decrease in AST activity was shown in vaccinated birds fed a diet with increased CP in comparison with vaccinated ones receiving a diet with normative CP, although this activity was still higher than in unvaccinated birds receiving dietary normative CP. In vaccinated birds, the supplementation with HE resulted in decrease in AST activity to the level found in unvaccinated birds fed with unsupplemented diet. A significant increase in AST activity was observed in birds receiving the diet with increased CP and supplemented with HE in comparison with those fed an unsupplemented diet with increased CP, while in birds receiving normative dietary CP, the HE supplementation decreased the AST activity. Generally, the effects of increased CP or HE supplementation were observed for ALT activity. The activity of this enzyme was reduced in birds fed a diet with increased CP, but elevated in birds supplemented with HE. An almost two-fold increase in ALT activity was observed in unvaccinated birds as a result of HE supplementation, although the effect of the vaccination on ALT activity was not significant. LDH activity was decreased in vaccinated birds, while dietary HE supplementation resulted in an increase of LDH activity (Table 4, Table S3 in Supplementary Materials).

In regard to lipid parameters (Table 5, Table S4 in Supplementary Materials), ACV significantly increased HDL-C and decreased LDL-C levels. Supplementation with HE reduced TG levels and increased HDL-C levels; however, these effects were significant only in unvaccinated chickens.

Glucose level was significantly decreased in vaccinated birds, while TP or UA levels were not affected by any of the experimental factors (Table 6). The CREAT concentration was decreased in birds receiving increased dietary CP levels, while HE supplementation resulted in increased BIL levels (Table 6, Table S4 in Supplementary Materials).

In regard to the parameters describing redox status (Table 7, Table S6 in Supplementary Materials), ACV increased CAT activity and FRAP values, and reduced blood MDA concentration. Generally, increased CP significantly elevated FRAP and SOD activity, but reduced LOOH concentration. For CAT activity, a significant ACV × CP × HE interaction was observed. CAT activity was nearly two-fold higher in vaccinated chickens receiving diets with an increased CP level without HE supplementation, or 1.5-times higher in those receiving the low CP level diets with HE addition, compared to the group of vaccinated untreated chickens, while the combined use of increased CP or HE supplementation had no significant effects. In unvaccinated birds receiving increased dietary CP an increase in FRAP was found in comparison with birds fed with normative CP. In vaccinated ones receiving increased CP significantly lower FRAP was observed in comparison with those fed a diet with normative CP, although this result was still significantly increased in comparison with unvaccinated birds fed a diet with normative CP level. HE supplementation increased FRAP, but only in unvaccinated birds.

Increased CP significantly reduced LOOH concentration in birds vaccinated against coccidiosis, but elevated MDA concentration in unvaccinated birds.

## 4. Discussion

Data describing blood variables in chickens vaccinated against coccidiosis are limited; therefore, the authors decided to discuss many of the obtained results with reports on the impact of coccidiosis. Moreover, in some studies multiple doses of ACV have been used to induce subclinical coccidiosis in broilers [22].

The obtained hematological values for 14-day-old chickens were surprising; significant reductions of WBC counts and lymphocytes proportion in leukograms as a result of the vaccination were observed. ACV also led to an increased proportion of heterophils in leukograms. Heterophils are the predominant granulated leukocytes in acute inflammatory responses in gallinaceous birds. An increase in heterophils is usually associated with their participation in the initial response during the first 6 to 12 h following an immunological challenge [23,24]. Ognik et al. [25] identified an increase in the number of heterophils and a decrease in the number of lymphocytes as a result of acute infections, for example coccidiosis, along with severe stress. In the current study, the prolonged (up to the 14th day after the vaccination) higher proportion of H and higher H/L ratio could suggest inflammation and increased stress levels in birds vaccinated against coccidiosis. The positive effects of increased dietary CP or HE supplementation in chickens vaccinated against coccidiosis such as increase of lymphocytes and reduction of heterophils proportion in leukograms, as well as a consequently reduced H/L ratio indicate the beneficial effect of these factors on the alleviation of post-vaccination stress. In contrast with these results, da Silva et al. [24] reported reduced Ht and Hb values, along with increased leukocyte and lymphocyte counts, in 34-day-old chickens vaccinated at 3 d of age, but not statistically significant effect of vaccination on H/L ratio was observed.

Coccidiosis often leads to the induction of non-specific immune responses with the production of reactive nitrogen species such as nitric oxide (NO) and reactive oxygen species (ROS) [26,27]. This mechanism is primarily explained as a response of the organism (production of reactive radical species against oocysts), but this may also negatively affect chickens if their antioxidant defense system is not able to protect them against oxidative damage. Some reports indicate the impairment of the anti-oxidant/pro-oxidant equilibrium in favor of pro-oxidants, indicating oxidative stress as a result of this poultry parasitosis [28,29,30]. In broilers infected with *E. acervulina* [30] and *E. tenella* [31,32], oxidative stress was observed as an effect of the increased NO and/or MDA concentration and alterations in the activity of anti-oxidant enzymes, i.e., reduced SOD and increased CAT activity. In the current study, the influence of ACV on redox status was reflected in a reduced concentration of MDA, and increased CAT activity, without affecting SOD activity. Presumably, permanent contact with low doses of attenuated oocysts led to a different adaptive mechanism which, in the case of enzymatic anti-oxidants, relied on the stimulation of CAT rather than SOD activity. The oxidative/anti-oxidative parameters obtained in the current study could not clearly confirm the presence of oxidative stress in vaccinated birds, although a higher proportion of heterophils was noted in leukograms. The increase in the level of heterophils, whose mode of action in the mechanism of innate non-specific immunity response is to generate ROS in the oxidative burst, results in increased LOOH and/or MDA content. In the current study, the profiles of pro-oxidative/anti-oxidative parameters, such as LOOH and MDA contents influenced by ACV, were not consistent with those expected. It is possible that this can be partially explained by the results of phagocytic assays which indicate the suppression of the phagocytic activity of leukocytes in birds vaccinated against coccidiosis (reduced %PC, PI, NBT). The same effect was noted in terms of the impact of HE. This inhibitory effect of HE on the function of phagocytic cells is difficult to explain. It was expected that the *Echinacea purpurea* extract included in HE would exert immuno-stimulatory properties, as Allen [33] has proposed this herb as a potential adjuvant for live vaccines against coccidiosis.

The concentration of serum lysozyme, a component of humoral non-specific immunity, was significantly decreased by ACV vaccination or HE supplementation. Such a reducing effect of ACV can be directly attributed to the negative effect of the presence and multiplication of intestinal oocysts, regarded as a challenge similar to infection. Sotirov and Koinarski [34] observed decreased serum lysozyme concentrations in chickens infected with *E. tenella*, along with increased cecal lysozyme levels (8 to 78 times higher than in control groups); thus indicating the direction of lysozyme mobilization. In the current study, the concentration of serum lysozyme was about 35% lower in chickens vaccinated against coccidiosis, indicating that the period of vaccination +14 d was not sufficient for a return to homeostasis. The immuno-suppressive effect of HE in respect to lysozyme concentration was, again, unexpected and requires further verification.

Generally, the GLU level recorded in this study was found to be reduced in vaccinated chickens. This effect could be attributed to inflammation in the intestinal tract followed by glucose absorption [35] being affected as a direct result of impaired gut integrity.

Analysis of AST, ALT or LDH activities is generally used to evaluate the effect of various feed supplements on the liver profile of birds. A lack of effect on, or decrease in, AST, ALT or LDH activities indicates that the supplement does not interfere with liver function [29]. While ACV significantly increased AST activity, increased CP or supplementation of HE significantly reduced it in vaccinated birds, indicating the hepatoprotective effect of these treatments in broilers vaccinated against coccidiosis. Increased AST and ALT activities as a result of coccidiosis have been reported in previous studies by Patra et al. [36] and Abd El-Maksoud et al. [35].

Abd El-Maksoud et al. [35] also investigated the effect of the infection of hens with coccidiosis on lipid parameters and indicated a significant effect of parasitosis observed as a reduction in TC, TG, HDL-C and LDL-C fractions compared to a non-infected group. In the current study, we found a higher HDL-cholesterol fraction in vaccinated compared to non-vaccinated birds. HE supplementation also increased HDL-cholesterol fraction, a known effect of botanicals [37,38,39].

## 5. Conclusions

In conclusion, the effect of increased CP level and/or dietary supplementation with herbal extracts, along with the impact of live ACV, is not clear. At the same time, vaccination with live oocysts is regarded as a challenge, and causes similar effects to mild subclinical coccidiosis, frequently affecting birds’ responses in a different manner from their responses to clinical coccidiosis. In spite of these inconsistent results, the negative effect of ACV was generally reflected in many of the studied hematological, immunological, biochemical and redox blood parameters. The application of dietary treatments, such as increased CP or HE supplementation, could be recommended due to their positive effect in terms of inducing: reduced H/L ratios, a signal of lowered stress; and lowered AST, a parameter demonstrating hepatoprotective properties. Moreover, increased dietary CP resulted in increased CAT activity or decreased LOOH content in vaccinated birds, indicators of the activation of antioxidant activity. However, the simultaneous use of these nutritional methods offered no additional relevant benefits in terms of most of the blood indices of vaccinated chickens.

## Figures and Tables

**Table 1:** Composition of experimental diet for starter (1–21 d) phase and nutrient content in basal feed mixtures.

Ingredient [g/kg]:	Normative CP Level	Increased CP Level
Maize	579.3	505.3
Soybean meal	360	420
Soybean oil	18	32
Limestone	16	16
Monocalcium phosphate	14.5	14.5
Sodium choloride	3	3
dl-Methionine	2	2
l-Lysine hydrochloride	1.2	1.2
Vitamin-mineral premix ^1^	6	6
Calculated nutritional value per kg of feed:		
Crude protein [g/kg]	216	236
Metabolizable energy [MJ/kg]	12.3	12.3
Analysed chemical composition of the basal feed mixtures [g/kg]:		
Dry matter	874	877
Crude ash	69	76.1
Crude protein	213.3	232.4
Crude fat	35.1	36.1
Crude fibre	24.2	22.9
Calcium	14.7	15.9
Phosphorus	7.45	7.33
Asp [g/kg]	21.86	25.31
Tre [g/kg]	7.7	8.65
Ser [g/kg]	10.21	11.23
Glu [g/kg]	36.95	41.17
Pro [g/kg]	12.11	13.17
Gli [g/kg]	8.51	9.67
Ala [g/kg]	10.05	11.19
Val [g/kg]	9.75	11.06
Ile [g/kg]	8.68	10.01
Leu [g/kg]	17.49	19.6
Tyr [g/kg]	6.73	7.25
Fen [g/kg]	11.32	12.6
His [g/kg]	6.41	7.25
Lis [g/kg]	14.54	16.37
Arg [g/kg]	15.68	17.58
Cys [g/kg]	3.11	3.27
Met [g/kg]	5.11	5.46
Trp [g/kg]	2.6	2.82

^1^ The premix provided, per 1 kg of diet vitamin A—12,000 IU; vitamin D3—3000 IU; vitamin E—42 IU; vitamin K3—3.6 mg; vitamin B1—2.4 mg; vitamin B2—8.4 mg; vitamin B6—6 mg; vitamin B12 –0.048 mg; Ca-pantotenate—12 mg; niacine—48 mg; folic acid—1.2 mg; biotin—0.096 mg; choline chloride—177 mg; manganese—96 mg; zinc—72 mg; iron—72 mg; copper—18 mg; iodine—2.4 mg; selenium—0.3 mg.

**Table 2:** The hematological indices and leukogram of chicken blood collected at 14 d of age.

Factors			RBC	WBC	Ht	Hb	H	L	MONO	EOS	BASO	H/L
ACV	CP	HE	[10^12^ L^−1^]	[10^9^ L^−1^]	[L L^−1^]	[g^−1^]	[%]	[%]	[%]	[%]	[%]	
**−**	**N**	**−**	2.20	15.9	27.0	6.28	27.7	69.3	1.00	1.00	1.00	0.40
		**+**	2.68	17.6	29.6	5.38	33.8	62.2	1.50	1.00	1.50	0.55
	**I**	**−**	2.44	22.3	27.9	4.45	32.0	64.3	1.67	0.83	1.17	0.50
		**+**	2.90	18.6	27.5	5.47	29.3	67.5	1.17	0.83	1.17	0.43
**+**	**N**	**−**	2.45	15.1	29.2	6.80	42.3	53.8	1.67	1.00	1.17	0.79
		**+**	2.60	14.8	28.2	4.97	40.2	55.8	1.33	1.17	1.50	0.72
	**I**	**−**	2.64	13.6	28.2	5.90	32.0	64.8	1.00	1.00	1.17	0.51
		**+**	2.64	13.2	27.2	5.65	26.8	70.0	1.17	0.83	1.17	0.38
**SEM**			0.057	0.689	0.469	0.336	0.879	0.949	0.112	0.098	0.098	0.022
**Significance (*p*-Value)**												
**Effects**		**ACV**	NS	*	NS	NS	NS	*	NS	*	NS	*
		**CP**	NS	NS	NS	NS	NS	*	NS	NS	NS	*
		**HE**	NS	NS	NS	NS	NS	NS	NS	NS	NS	NS
**Interactions**		**ACV × CP**	NS	*	NS	NS	NS	*	NS	NS	NS	*
		**ACV × HE**	NS	NS	NS	NS	NS	*	NS	NS	NS	*
		**CP × HE**	NS	*	NS	NS	NS	*	NS	NS	NS	*
		**ACV × CP × HE**	NS	*	NS	NS	NS	NS	NS	NS	NS	NS

NS—*p* > 0.05; *—*p* ≤ 0.05; VAC—anticoccidial vaccine; CP—crude protein level, N—normative; I—increased; HE—herbal extract blend; SEM—standard errors mean; RBC—red blood cells; WBC—white blood cells; Ht—haematocrit; Hb—haemoglobin; H—heterophils; L—lymphocytes; MONO—monocytes; EOS—eosinophils; BASO—basophils; H/L—heterophils/lymphocytes.

**Table 3:** The immunological indices of chicken blood collected at 14 d of age.

Factors			Lysozyme [mg L^−1^]	%PC	PI	NBT: Positive Heterophils
ACV	CP	HE				[%]
**−**	**N**	**−**	3.79	44.0	5.95	26.3
		**+**	2.81	36.3	5.90	23.8
	**I**	**−**	3.53	45.8	6.40	29.0
		**+**	2.56	38.8	6.00	22.8
**+**	**N**	**−**	2.30	36.3	5.72	23.7
		**+**	1.50	32.5	5.55	19.2
	**I**	**−**	2.51	36.8	5.95	22.8
		**+**	1.89	33.7	5.52	20.7
**SEM**			0.138	1.124	0.087	0.738
**Significance (*p*-Value)**						
**Effects**		**ACV**	*	*	*	*
		**CP**	NS	NS	NS	NS
		**HE**	*	*	NS	*
**Interactions**		**ACV × CP**	NS	NS	NS	NS
		**ACV × HE**	NS	NS	NS	NS
		**CP × HE**	NS	NS	NS	NS
		**ACV × CP × HE**	NS	NS	NS	NS

NS—*p* > 0.05; *—*p* ≤ 0.05; VAC—anticoccidial vaccine; CP—crude protein level, N—normative; I—increased; HE—herbal extract blend; SEM—standard errors mean; %PC—percentage of phagocytic cells; PI—phagocytic index; NBT—test reduction of nitroblue tetrazolium.

**Table 4:** The biochemical indices of chicken blood collected at 14 d of age.

Factors			AST [U/L]	ALT [U/L]	LDH [U/L]
**ACV**	**CP**	**HE**			
**−**	**N**	**−**	233	6.68	494
		**+**	236	10.2	803
	**I**	**−**	330	4.02	466
		**+**	418	9.54	534
**+**	**N**	**−**	432	8.98	292
		**+**	335	9.13	283
	**I**	**−**	292	8.65	264
		**+**	303	6.29	404
**SEM**			11.79	0.445	34.6
**Significance (*p*-Value)**					
**Effects**		**ACV**	*	NS	*
		**CP**	NS	*	NS
		**HE**	NS	*	*
**Interactions**		**ACV × CP**	*	NS	NS
		**ACV × HE**	*	*	NS
		**CP × HE**	*	NS	NS
		**ACV × CP × HE**	NS	NS	NS

NS—*p* > 0.05; *—*p* ≤ 0.05; VAC—anticoccidial vaccine; CP—crude protein level, N—normative; I—increased; HE—herbal extract blend; SEM—standard errors mean; AST—aspartate aminotransferase; ALT—alanine aminotransferase; LDH—lactate dehydrogenase.

**Table 5:** The lipid indices of chicken blood collected at 14 d of age.

Factors			TG [mmol/L]	TC [mmol/L]	HDL-C [mmol/L]	LDL-C [mmol/L]
**ACV**	**CP**	**HE**				
**−**	**N**	**−**	0.397	2.25	1.11	0.466
		**+**	0.174	2.82	1.64	0.459
	**I**	**−**	0.321	2.28	1.13	0.379
		**+**	0.215	2.61	1.64	0.347
**+**	**N**	**−**	0.311	2.48	1.66	0.233
		**+**	0.321	2.39	1.49	0.260
	**I**	**−**	0.262	2.51	1.36	0.406
		**+**	0.277	2.44	1.60	0.241
**SEM**			0.017	0.049	0.046	0.030
**Significance (*p*-Value)**						
**Effects**		**ACV**	NS	NS	*	*
		**CP**	NS	NS	NS	NS
		**HE**	*	NS	*	NS
**Interactions**		**ACV × CP**	NS	NS	NS	NS
		**ACV × HE**	*	*	*	NS
		**CP × HE**	NS	NS	NS	NS
		**ACV × CP × HE**	NS	NS	NS	NS

NS—*p* > 0.05; *—*p* ≤ 0.05; VAC—anticoccidial vaccine; CP—crude protein level, N—normative; I—increased; HE—herbal extract blend; SEM—standard errors mean; TG—triacylglycerol; TC—total cholesterol; HDL-C—high-density lipoprotein cholesterol; LDL-C—low-density lipoprotein cholesterol.

**Table 6:** The biochemical indices chicken blood collected at 14 d of age.

Factors			TP [g/L]	GLU [mmol/L]	UA [µmol/L]	CREAT [µmol/L]	BIL [µmol/L]
**ACV**	**CP**	**HE**					
**−**	**N**	**−**	29.5	19.2	215	21.2	28.4
		**+**	31.8	18.0	341	26.0	30.8
	**I**	**−**	27.7	16.5	298	19.2	28.4
		**+**	29.8	17.3	259	17.8	30.4
**+**	**N**	**−**	29.6	14.6	352	20.5	28.7
		**+**	29.6	15.8	302	23.3	29.4
	**I**	**−**	30.2	13.3	289	21.2	29.5
		**+**	29.8	17.0	281	19.2	35.6
**SEM**			0.511	0.511	15.94	5.194	0.699
**Significance (*p*-Value)**							
**Effects**		**ACV**	NS	*	NS	NS	NS
		**CP**	NS	NS	NS	*	NS
		**HE**	NS	NS	NS	NS	*
**Interactions**		**ACV × CP**	NS	NS	NS	NS	NS
		**ACV × HE**	NS	NS	NS	NS	NS
		**CP × HE**	NS	NS	NS	NS	NS
		**ACV × CP × HE**	NS	NS	NS	NS	NS

NS—*p* > 0.05; *—*p* ≤ 0.05; VAC—anticoccidial vaccine; CP—crude protein level, N—normative; I—increased; HE—herbal extract blend; SEM—standard errors mean; TP—total protein; GLU—glucose; UA—uric acid; CREAT—creatinine; BIL—bilirubin.

**Table 7:** The redox status indices of chicken blood collected at 14 d of age.

Factors			FRAP [μmol/L]	SOD [U/mL]	CAT [U/mL]	LOOH [μmol/L]	MDA [μmol/L]
**ACV**	**CP**	**HE**					
**−**	**N**	**−**	98	27.2	31.3	1.98	0.842
		**+**	292	26.5	28.6	2.15	0.657
	**I**	**−**	291	25.5	19.7	1.44	0.925
		**+**	532	29.0	20.4	1.91	1.345
**+**	**N**	**−**	459	28.6	38.2	3.06	0.793
		**+**	395	25.3	52.9	1.99	0.688
	**I**	**−**	363	29.5	76.0	1.18	0.570
		**+**	330	29.7	35.2	1.92	0.556
**SEM**			19.78	0.417	2.975	0.111	0.058
**Significance (*p*-Value)**							
**Effects**		**ACV**	*	NS	*	NS	*
		**CP**	*	*	NS	*	NS
		**HE**	*	NS	*	NS	NS
**Interactions**		**ACV × CP**	*	NS	*	*	*
		**ACV × HE**	*	NS	NS	NS	NS
		**CP × HE**	NS	*	*	*	NS
		**ACV × CP × HE**	NS	NS	*	NS	NS

NS—*p* > 0.05; *—*p* ≤ 0.05; VAC—anticoccidial vaccine; CP—crude protein level, N—normative; I—increased; HE—herbal extract blend; SEM—standard errors mean; FRAP—total antioxidant potential, as a ferric reducing ability of plasma; SOD—superoxide dismutase; CAT—catalase; LOOH—lipid peroxides; MDA—malondialdehyde.

## Supplementary Materials

The following are available online at http://www.mdpi.com/2076-2615/8/11/208/s1.
