# Supplementary material for: Effect of Dietary Crude Protein Level and Supplemental Herbal Extract Blend on Selected Blood Variables in Broiler Chickens Vaccinated against Coccidiosis
Source: Animals (Basel). 2018 Nov 15;8(11):208. doi: 10.3390/ani8110208 (PMC6262617; doi:10.3390/ani8110208)
Supplement: Supplementary file 1 [file animals-08-00208-s001.docx]

**Table S1.** The hematological indices and leukogram of chicken blood collected at 14 d of age.

| **Main and interaction effects** | **RBC [10^12^ l^−1^]** | **WBC [10^9^ l^−1^]** | **Ht [l l^−1^]** | **Hb [g l^−1^]** | **H [%]** | **L [%]** | **MONO [%]** | **EOS [%]** | **BASO [%]** | **H/L** |
| --- | --- | --- | --- | --- | --- | --- | --- | --- | --- | --- |
| **ACV** |  |  |  |  |  |  |  |  |  |  |
| UNVAC | 2.55 | 18.6 ^a^ | 28.0 | 5.39 | 30.7 ^a^ | 65.8 ^a^ | 1.33 | 0.917 | 1.21 | 0.473 ^a^ |
| VAC | 2.58 | 14.1 ^b^ | 28.2 | 5.83 | 35.3 ^b^ | 61.1 ^b^ | 1.29 | 1.000 | 1.25 | 0.599 ^b^ |
| **CP** |  |  |  |  |  |  |  |  |  |  |
| CPN | 2.48 | 15.8 | 28.5 | 5.86 | 36.0 ^a^ | 60.3 ^a^ | 1.38 | 1.042 | 1.29 | 0.615 ^a^ |
| CPI | 2.66 | 16.9 | 27.7 | 5.37 | 30.0 ^b^ | 66.7 ^b^ | 1.25 | 0.875 | 1.17 | 0.457 ^b^ |
| **HE** |  |  |  |  |  |  |  |  |  |  |
| HE- | 2.43 ^a^ | 16.7 | 28.1 | 5.86 | 33.5 | 63.1 | 1.33 | 0.958 | 1.13 | 0.549 |
| HE+ | 2.71 ^b^ | 16.0 | 28.1 | 5.37 | 32.5 | 63.9 | 1.29 | 0.958 | 1.33 | 0.523 |
| **ACV x CP** |  |  |  |  |  |  |  |  |  |  |
| UNVAC x CPN | 2.44 | 16.7 ^b^ | 28.3 | 5.83 | 30.8 ^b^ | 65.8 ^a^ | 1.25 | 1.000 | 1.25 | 0.477 ^b^ |
| UNVAC x CPI | 2.67 | 20.4 ^a^ | 27.7 | 4.96 | 30.7 ^b^ | 65.9 ^a^ | 1.42 | 0.833 | 1.17 | 0.470 ^b^ |
| VAC x CPN | 2.52 | 14.9 ^b,c^ | 28.7 | 5.89 | 41.3 ^a^ | 54.8 ^b^ | 1.50 | 1.083 | 1.33 | 0.754 ^a^ |
| VAC x CPI | 2.64 | 13.4 ^c^ | 27.7 | 5.77 | 29.4 ^b^ | 67.4 ^a^ | 1.08 | 0.917 | 1.17 | 0.444 ^b^ |
| **ACV x HE** |  |  |  |  |  |  |  |  |  |  |
| UNVAC x HE - | 2.32 | 19.1 | 27.5 | 5.36 | 29.8 ^b^ | 66.8 ^b^ | 1.33 | 0.917 | 1.08 | 0.452 ^c^ |
| UVVAC x HE+ | 2.79 | 18.1 | 28.5 | 5.43 | 31.6 ^b,c^ | 64.8 ^b,c^ | 1.33 | 0.917 | 1.33 | 0.494 ^b,c^ |
| VAC x HE- | 2.55 | 14.3 | 28.7 | 6.35 | 37.2 ^a^ | 59.3 ^a^ | 1.33 | 1.000 | 1.17 | 0.647 ^a^ |
| VAC x HE+ | 2.62 | 14.0 | 27.7 | 5.31 | 33.5 ^c^ | 62.9 ^c^ | 1.25 | 1.000 | 1.33 | 0.552 ^b^ |
| **CP x HE** |  |  |  |  |  |  |  |  |  |  |
| CPN x HE - | 2.32 | 15.5 ^b^ | 28.1 | 6.54 | 35.0 ^b^ | 61.6 ^b,c^ | 1.33 | 1.000 | 1.08 | 0.594 ^a^ |
| CPN x HE + | 2.64 | 16.2 ^a,b^ | 28.9 | 5.18 | 37.0 ^b^ | 59.0 ^b^ | 1.42 | 1.083 | 1.50 | 0.637 ^a^ |
| CPI x HE- | 2.54 | 17.9 ^a^ | 28.0 | 5.17 | 32.0 ^a^ | 64.6 ^c^ | 1.33 | 0.917 | 1.17 | 0.505 ^b^ |
| CPI x HE+ | 2.77 | 15.9 ^b^ | 27.3 | 5.56 | 28.1 ^c^ | 68.8 ^a^ | 1.17 | 0.833 | 1.17 | 0.409 ^c^ |
| **SEM** | 0.057 | 0.689 | 0.469 | 0.336 | 0.879 | 0.949 | 0.112 | 0.098 | 0.098 | 0.022 |

a, b, c, d means in columns with different superscripts differ significantly at *p* ≤ 0.05 for main effects and their interactions; ACV––anticoccidial vaccine; UNVAC––unvaccinated; VAC––vaccinated; CP––crude protein level, CPN––normative level of CP; CPI––increased level of CP; HE––herbal extract blend; RBC––red blood cells; WBC––white blood cells; Ht––haematocrit; Hb––haemoglobin; H––heterophils; L––lymphocytes; MONO––monocytes; EOS––eosinophils; BASO––basophils; H/L––heterophils/lymphocytes; SEM––standard errors mean.

**Table S2.** The immunological indices of chicken blood collected at 14 d of age.

| **Main and interaction effects** | **Lysozyme**  **[mg l^-1^]** | **%PC** | **PI** | **NBT: positive heterophils [%]** |
| --- | --- | --- | --- | --- |
| **ACV** |  |  |  |  |
| UNVAC | 3.17 ^a^ | 41.3 ^a^ | 6.06 ^a^ | 25.5 ^a^ |
| VAC | 2.05 ^b^ | 34.8 ^b^ | 5.68 ^b^ | 21.6 ^b^ |
| **CP** |  |  |  |  |
| CPN | 2.60 | 37.3 | 5.78 | 23.3 |
| CPI | 2.62 | 38.8 | 5.97 | 23.8 |
| **HE** |  |  |  |  |
| HE- | 3.03 ^a^ | 40.8 ^a^ | 6.00 | 25.5 ^a^ |
| HE+ | 2.19 ^b^ | 35.3 ^b^ | 5.74 | 21.6 ^b^ |
| **ACV x CP** |  |  |  |  |
| UNVAC x CPN | 3.30 | 40.2 | 5.93 | 25.1 |
| UNVAC x CPI | 3.04 | 42.3 | 6.20 | 25.9 |
| VAC x CPN | 1.90 | 34.4 | 5.63 | 21.4 |
| VAC x CPI | 2.20 | 35.3 | 5.73 | 21.8 |
| **ACV x HE** |  |  |  |  |
| UNVAC x HE - | 3.66 | 44.9 | 6.18 | 27.7 |
| UVVAC x HE+ | 2.68 | 37.6 | 5.95 | 23.3 |
| VAC x HE- | 2.40 | 36.6 | 5.83 | 23.3 |
| VAC x HE+ | 1.70 | 33.1 | 5.53 | 19.9 |
| **CP x HE** |  |  |  |  |
| CPN x HE - | 3.04 | 40.2 | 5.83 | 25.0 |
| CPN x HE + | 2.16 | 34.4 | 5.73 | 21.5 |
| CPI x HE- | 3.02 | 41.3 | 6.18 | 25.9 |
| CPI x HE+ | 2.22 | 36.3 | 5.76 | 21.8 |
| SEM | 0.138 | 1.124 | 0.087 | 0.738 |

a, b means in columns with different superscripts differ significantly at *p* ≤ 0.05 for main effects and their interactions; ACV––anticoccidial vaccine; UNVAC––unvaccinated; VAC––vaccinated; CP––crude protein level, CPN––normative level of CP; CPI––increased level of CP; HE––herbal extract blend; %PC––percentage of phagocytic cells; PI––phagocytic index; NBT––reduction of nitroblue tetrazolium; SEM––standard errors mean.

**Table S3.** The biochemical indices of chicken blood collected at 14 d of age.

| **Main and interaction effects** | **AST**  **[U/ l]** | **ALT**  **[U/ l]** | **LDH**  **[U/ l]** |
| --- | --- | --- | --- |
| **ACV** |  |  |  |
| UNVAC | 304 ^a^ | 7.60 | 574 ^a^ |
| VAC | 341 ^b^ | 8.26 | 311 ^b^ |
| **CP** |  |  |  |
| CPN | 309 | 8.74 ^a^ | 468 |
| CPI | 336 | 7.13 ^b^ | 417 |
| **HE** |  |  |  |
| HE- | 322 | 7.08 ^a^ | 379 ^a^ |
| HE+ | 323 | 8.79 ^b^ | 506 ^b^ |
| **ACV x CP** |  |  |  |
| UNVAC x CPN | 234 ^a^ | 8.43 | 649 |
| UNVAC x CPI | 373 ^c^ | 6.78 | 500 |
| VAC x CPN | 383 ^c^ | 9.06 | 288 |
| VAC x CPI | 297 ^b^ | 7.47 | 334 |
| **ACV x HE** |  |  |  |
| UNVAC x HE - | 281 ^a^ | 5.35 ^a^ | 480 |
| UVVAC x HE+ | 327 ^bc^ | 9.86 ^b^ | 669 |
| VAC x HE- | 362 ^c^ | 8.82 ^b^ | 278 |
| VAC x HE+ | 319 ^a,b^ | 7.71 ^b^ | 344 |
| **CP x HE** |  |  |  |
| CPN x HE - | 332 ^b,c^ | 7.83 | 393 |
| CPN x HE + | 285 ^a^ | 9.66 | 543 |
| CPI x HE- | 310 ^a,b^ | 6.34 | 365 |
| CPI x HE+ | 360 ^c^ | 7.92 | 469 |
| **SEM** | 11.79 | 0.445 | 34.6 |

a, b, c means in columns with different superscripts differ significantly at *p* ≤ 0.05 for main effects and their interactions; ACV––anticoccidial vaccine; UNVAC––unvaccinated; VAC––vaccinated; CP––crude protein level, CPN––normative level of CP; CPI––increased level of CP; HE––herbal extract blend; AST––aspartate aminotransferase; ALT––alanine aminotransferase; LDH––lactate dehydrogenase; SEM––standard errors mean.

**Table S4.** The lipid indices of chicken blood collected at 14 d of age.

| Main and interaction effects | TG  [mmol/l] | TC  [mmol/l] | HDL-C [mmol/l] | LDL-C  [mmol/l] |
| --- | --- | --- | --- | --- |
| **ACV** |  |  |  |  |
| UNVAC | 0.277 | 2.49 | 1.38 ^a^ | 0.413 ^a^ |
| VAC | 0.293 | 2.46 | 1.53 ^b^ | 0.285 ^b^ |
| **CP** |  |  |  |  |
| CPN | 0.301 | 2.49 | 1.47 | 0.355 |
| CPI | 0.269 | 2.46 | 1.43 | 0.343 |
| **HE** |  |  |  |  |
| HE- | 0.323 ^a^ | 2.38 | 1.31 ^a^ | 0.371 |
| HE+ | 0.247 ^b^ | 2.57 | 1.59 ^b^ | 0.327 |
| **ACV x CP** |  |  |  |  |
| UNVAC x CPN | 0.286 | 2.54 | 1.37 | 0.462 |
| UNVAC x CPI | 0.268 | 2.45 | 1.38 | 0.363 |
| VAC x CPN | 0.316 | 2.43 | 1.57 | 0.247 |
| VAC x CPI | 0.270 | 2.48 | 1.48 | 0.324 |
| **ACV x HE** |  |  |  |  |
| UNVAC x HE - | 0.359 ^b^ | 2.27 ^a^ | 1.12 ^a^ | 0.423 |
| UVVAC x HE+ | 0.195 ^a^ | 2.72 ^b^ | 1.64 ^b^ | 0.403 |
| VAC x HE- | 0.287 ^ab^ | 2.50 ^ab^ | 1.51 ^ab^ | 0.320 |
| VAC x HE+ | 0.299 ^b^ | 2.41 ^a^ | 1.55 ^a^ | 0.251 |
| **CP x HE** |  |  |  |  |
| CPN x HE - | 0.354 | 2.37 | 1.38 | 0.350 |
| CPN x HE + | 0.248 | 2.60 | 1.56 | 0.359 |
| CPI x HE- | 0.292 | 2.40 | 1.24 | 0.393 |
| CPI x HE+ | 0.246 | 2.53 | 1.62 | 0.294 |
| **SEM** | 0.017 | 0.049 | 0.046 | 0.030 |

a, b means in columns with different superscripts differ significantly at *p* ≤ 0.05 for main effects and their interactions; ACV––anticoccidial vaccine; UNVAC––unvaccinated; VAC––vaccinated; CP––crude protein level, CPN––normative level of CP; CPI––increased level of CP; HE––herbal extract blend; TG––triacylglycerol; TC––total cholesterol; HDL-C––high-density lipoprotein cholesterol; LDL-C––low-density lipoprotein cholesterol; SEM––standard errors mean.

**Table S5.** The biochemical indices chicken blood collected at 14 d of age.

| Main and interaction effects | TP  [g/l] | GLU [mmol/ l] | UA  [µmol/l] | CREAT [µmol/l] | BIL  [µmol/l] |
| --- | --- | --- | --- | --- | --- |
| **ACV** |  |  |  |  |  |
| UNVAC | 29.7 | 17.7 ^a^ | 278 | 21.0 | 29.5 |
| VAC | 29.7 | 15.2 ^b^ | 306 | 21.0 | 30.8 |
| **CP** |  |  |  |  |  |
| CPN | 29.8 | 16.9 | 303 | 22.8 ^a^ | 29.3 |
| CPI | 30.1 | 16.0 | 282 | 19.3 ^b^ | 31.0 |
| **HE** |  |  |  |  |  |
| HE- | 29.4 | 15.9 | 288 | 20.5 | 28.8 ^a^ |
| HE+ | 29.3 | 17.0 | 296 | 21.6 | 31.5 ^b^ |
| **ACV x CP** |  |  |  |  |  |
| UNVAC x CPN | 30.6 | 18.6 | 278 | 23.6 | 29.6 |
| UNVAC x CPI | 28.7 | 16.9 | 278 | 18.5 | 29.4 |
| VAC x CPN | 29.6 | 15.2 | 327 | 21.9 | 29.1 |
| VAC x CPI | 30.0 | 15.2 | 285 | 20.2 | 32.5 |
| **ACV x HE** |  |  |  |  |  |
| UNVAC x HE - | 28.6 | 17.8 | 256 | 20.2 | 28.4 |
| UVVAC x HE+ | 30.8 | 17.7 | 300 | 21.9 | 30.6 |
| VAC x HE- | 29.9 | 14.0 | 321 | 20.9 | 29.1 |
| VAC x HE+ | 29.7 | 16.4 | 292 | 21.2 | 32.5 |
| **CP x HE** |  |  |  |  |  |
| CPN x HE - | 29.5 | 16.9 | 284 | 20.9 | 28.5 |
| CPN x HE + | 30.7 | 16.9 | 322 | 24.6 | 30.1 |
| CPI x HE- | 29.0 | 14.9 | 293 | 20.2 | 29.0 |
| CPI x HE+ | 29.8 | 17.1 | 270 | 18.5 | 33.0 |
| **SEM** | 0.511 | 0.511 | 15.94 | 5.194 | 0.699 |

a, b means in columns with different superscripts differ significantly at *p* ≤ 0.05 for main effects and their interactions; ACV––anticoccidial vaccine; UNVAC––unvaccinated; VAC––vaccinated; CP––crude protein level, CPN––normative level of CP; CPI––increased level of CP; HE––herbal extract blend; TP––total protein; GLU––glucose; UA––uric acid; CREAT––creatinine; BIL––bilirubin; SEM––standard errors mean.

**Table S6.** The redox status indices of chicken blood collected at 14 d of age.

| **Main and interaction effects** | **FRAP μmol/l** | **SOD [U/ml]** | **CAT**  **[U/ml]** | **LOOH μmol/l** | **MDA μmol/l** |
| --- | --- | --- | --- | --- | --- |
| **ACV** |  |  |  |  |  |
| UNVAC | 303 ^a^ | 27.1 | 25.0 ^a^ | 1.87 | 0.942 ^a^ |
| VAC | 387 ^b^ | 28.3 | 50.6 ^b^ | 1.92 | 0.652 ^b^ |
| **CP** |  |  |  |  |  |
| CPN | 311 ^a^ | 26.9 ^a^ | 37.8 | 2.29 ^a^ | 0.745 |
| CPI | 379 ^b^ | 28.4 ^b^ | 37.8 | 1.49 ^b^ | 0.849 |
| **HE** |  |  |  |  |  |
| HE- | 303 ^a^ | 27.7 | 41.3 ^a^ | 1.91 | 0.783 |
| HE+ | 387 ^b^ | 27.6 | 34.3 ^b^ | 1.87 | 0.812 |
| **ACV x CP** |  |  |  |  |  |
| UNVAC x CPN | 195 ^a^ | 26.9 | 29.9 ^a^ | 2.06 ^a,c^ | 0.750 ^a^ |
| UNVAC x CPI | 411 ^c^ | 27.2 | 20.0 ^b^ | 1.67 ^b,c^ | 1.135 ^b^ |
| VAC x CPN | 427 ^c^ | 26.9 | 45.6 ^c^ | 2.53 ^a^ | 0.741 ^a^ |
| VAC x CPI | 347 ^b^ | 29.6 | 55.6 ^d^ | 1.31 ^b^ | 0.563 ^a^ |
| **ACV x HE** |  |  |  |  |  |
| UNVAC x HE - | 195 ^a^ | 26.4 | 25.5 | 1.71 | 0.884 |
| UVVAC x HE+ | 412 ^b^ | 27.7 | 24.5 | 2.03 | 1.001 |
| VAC x HE- | 411 ^b^ | 29.0 | 57.1 | 2.12 | 0.681 |
| VAC x HE+ | 363 ^b^ | 27.5 | 44.1 | 1.71 | 0.622 |
| **CP x HE** |  |  |  |  |  |
| CPN x HE - | 279 | 27.9 ^a,b^ | 34.7 ^b,c^ | 2.52 ^a^ | 0.818 |
| CPN x HE + | 343 | 25.9 ^b^ | 40.8 ^ab^ | 2.07 ^a,c^ | 0.673 |
| CPI x HE- | 327 | 27.5 ^ab^ | 47.8 ^a^ | 1.31 ^b^ | 0.748 |
| CPI x HE+ | 431 | 29.3 ^a^ | 27.8 ^c^ | 1.67 ^bc^ | 0.951 |
| **SEM** | 19.78 | 0.417 | 2.975 | 0.111 | 0.058 |

a, b, c, d means in columns with different superscripts differ significantly at *p* ≤ 0.05 for main effects and their interactions; ACV––anticoccidial vaccine; UNVAC––unvaccinated; VAC––vaccinated; CP––crude protein level, CPN––normative level of CP; CPI––increased level of CP; HE––herbal extract blend; FRAP––total antioxidant potential, as a ferric reducing ability of plasma; SOD––superoxide dismutase; CAT––catalase; LOOH––lipid peroxides; MDA––malondialdehyde; SEM––standard errors mean.
